# Oral delivery of stabilized lipid nanoparticles for nucleic acid therapeutics

**DOI:** 10.1007/s13346-024-01709-4

**Published:** 2024-09-19

**Authors:** Kanika Suri, Liam Pfeifer, Donna Cvet, Angela Li, Michael McCoy, Amit Singh, Mansoor M. Amiji

**Affiliations:** 1 Takeda Development Center Americas, Cambridge, MA USA; 2 Department of Bioengineering, College of Engineering, Northeastern University, Boston, MA USA; 3 Department of Pharmaceutical Sciences, Bouve College of Health Sciences, Northeastern University, Boston, MA USA; 4 Department of Chemical Engineering, College of Engineering, Northeastern University, Boston, MA USA

**Keywords:** Lipid Nanoparticles, Small Interfering RNA, Messenger RNA, And Oral Delivery

## Abstract

Gastrointestinal disorders originate in the gastrointestinal tract (GIT), and the therapies can benefit from direct access to the GIT achievable through the oral route. RNA molecules show great promise therapeutically but are highly susceptible to degradation and often require a carrier for cytoplasmic access. Lipid nanoparticles (LNPs) are clinically proven drug-delivery agents, primarily administered parenterally. An ideal Orally Delivered (OrD) LNP formulation should overcome the diverse GI environment, successfully delivering the drug to the site of action. A versatile OrD LNP formulation has been developed to encapsulate and deliver siRNA and mRNA in this paper. The formulations were prepared by the systematic addition of cationic lipid to the base LNP formulation, keeping the total of cationic lipid and ionizable lipid to 50 mol%. Biorelevant media stability depicted increased resistance to bile salt mediated destabilization upon the addition of the cationic lipid, however the in vitro efficacy data underscored the importance of the ionizable lipid. Based on this, OrD LNP was selected comprising of 20% cationic lipid and 30% ionizable lipid. Further investigation revealed the enhanced efficacy of OrD LNP in vitro after incubation in different dilutions of fasted gastric, fasted intestinal media, and mucin. Confocal imaging and flow cytometry confirmed uptake while in vivo studies demonstrated efficacy with siRNA and mRNA as payloads. Taken together, this research introduces OrD LNP to deliver nucleic acid locally to the GIT.

**Graphical Abstract:**

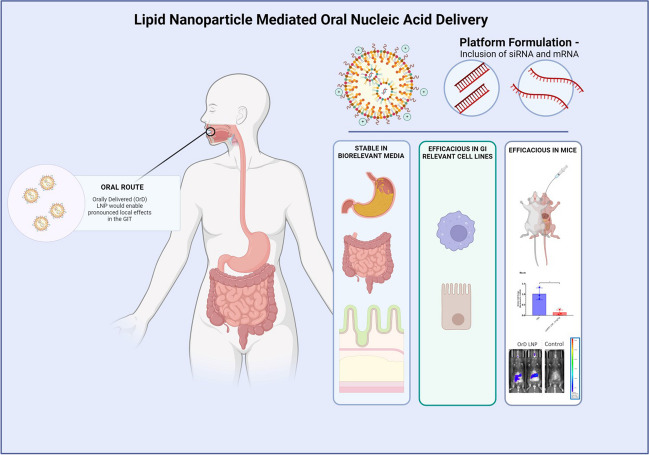

**Supplementary Information:**

The online version contains supplementary material available at 10.1007/s13346-024-01709-4.

## Introduction

Drug delivery vehicles bolster the therapeutic prowess of nucleic acid payloads. They offer protection to enzymes and other degradants, improve the half-life of the payload, and modulate the biodistribution, offering tissue and cell-specificity. Almost all the nucleic acids that have been translated into the clinic are administered parenterally through infusions or injections [1]. This approach is logical within the drug development process, particularly when establishing proof of concept or introducing novel modalities and mechanisms of action. Patient-centric considerations, along with other formulation attributes such as extended shelf-life, are typically prioritized only after safety and efficacy benchmarks have been achieved. Gastrointestinal (GI) ailments have origins in the GI tract (GIT), such as Eosinophilic Esophagitis in the esophagus [2], Gastroparesis in the stomach [3], Celiac disease in the small intestine [4], colorectal cancer in the colon [5] and IBD manifests itself differently through the GI tract with ulcerative colitis localized in the colon, and Crohn’s disease affecting the entire GIT [6]. Furthermore, any off-target effects of the therapy can be circumvented if administered locally. This accentuates the necessity for site-specific therapeutic approaches to gastrointestinal maladies. Consequently, it is unsurprising that the oral administration of macromolecules represents the quintessential objective that drug delivery scientists endeavor to realize. Oral delivery offers high patient compliance and lowers the burden on hospitals. The successful localized delivery of potent molecules offers targeted relief and reduces off-target effects. Furthermore, the GIT offers access to a wide variety of immune cells, providing a lucrative target for immune-modulatory therapies. Lastly, the vascularized nature of the GIT, provides access to systemic delivery of the payload.

Nucleic acids are extremely labile, and susceptible to degradation via nucleases abundant in the GIT [7]. Nucleic acids need to be protected for their safe transit from the oral route to the cytoplasm of GI cells. Nanoparticles have been evaluated for oral delivery of nucleic acids, generally are made up of polymers of natural proteins and carbohydrates [8–10]. Out of all the polymeric systems, hybrid systems can particularly provide multiple levels of protection and retain the integrity of the payload through the GIT. Successful polymeric hybrid systems used for oral delivery of nucleic acids include the nanoparticle in microsphere (NiMOS) that protects and delivers the nucleic acid locally. NiMOS are composed of multiple gelatin nanoparticles (GNPs) with encapsulated nucleic acid which are encased within a protective microparticle made up of a polymer such as poly(epsilon-caprolactone) (PCL) [11]. PCL protects the GNPs in the stomach, and degrades in response to the lipases and proteases present in the small and large intestines [11]. NiMOS deliver the payload locally and do not get systemically absorbed as was shown by In(111)-labeled GNPs [12], and have been shown to be more effective in transfecting luminal enterocytes [13]. NiMOS are versatile, having shown to alleviate inflammation with various payloads such as IL-10 pDNA in TNBS induced colitis model [13], with siTNFα and siCyD1 in DSS induced acute colitis model [14, 15], and siIL15 and siTG2 in Poly(I:C) model of celiac disease [16, 17]. NiMOS are prime example of designing orally delivered nucleic acid systems keeping the GI physiology in mind.

While valuable insights can be drawn from the use of polymeric vehicles, the success of orally administered LNPs has thus far been constrained. LNPs are the most potent form of nucleic acid transfection agents that have demonstrated clinical success. Major efforts have been made to improve their potency when administered parenterally. Typical LNP formulations that were originally developed for parenteral administration when used for oral delivery was met with varying degrees of success [18, 19]. Ball et al. explored the stability of their LNP formulation, 306O13 Lipidoid, under various gastrointestinal tract (GIT) conditions. The formulation comprised 306O13 Lipidoid: DSPC: Chol: PEG(2 k) = 50: 10: 38.5: 1.5 (mol%), with a mass ratio of ionizable lipid to siRNA at 5:1. It maintained encapsulation across a pH range from 2 to 7. However, challenges arose when pepsin led to aggregation and bile salts reduced its in vitro efficacy. Additionally, the encapsulated Cy5.5 labeled siRNA showed a very low signal, which might be attributed to either the low dose of 0.5 mg/kg or the label detaching from the siRNA. The study also noted that siGAPDH did not exhibit efficacy compared to untreated controls, and rectal LNP delivery at 5 mg/kg highlighted some limitations of the formulation in achieving successful transfection. In another study, El-Mayta et al. utilize barcode DNA encapsulated LNP composed of C12-200: DSPC: Chol: PEG (1 k) = 40:10:30:20 (mol%), with a mass ratio of ionizable lipid to barcode DNA of 10: 1. This LNP accumulated the most in the small intestine and the colon. While this study utilizes DNA as the payload and deep sequencing to depict accumulation, efficacy would be predicated on the successful protein production which this study does not particularly evaluate. As such these studies illustrate some limitations and urge further investigation into formulation development to balance GIT stability and efficacy.

In the current research, our main aim was to develop an oral LNP platform that can deliver a variety of nucleic acid payloads. We chose C12-200 as the ionizable lipid since it lacks hydrolysable bonds and has demonstrated high transfection efficiency for siRNA as well as mRNA delivery [20, 21]. Additionally, DOTMA (N-[1-(2,3-dioleyloxy)propyl]-N,N,N-trimethylammonium chloride) was selected as permanently charged cationic lipid to increase the core positive charge for better nucleic acid complexation, which would impart increased stability as LNP traverses through the GIT. We demonstrated that addition of DOTMA to the C12-200 LNPs improved stability in biorelevant media and exhibited efficacy with siRNA (or mRNA) in vitro as well as in vivo. The incorporation of 20% DOTMA into the formulation completed the makeup of components for our candidate orally delivered (OrD) LNP. Our results validate OrD LNPs as a platform technology for delivery of variety of nucleic acid payloads with potential for rapid clinical translation to the clinic.

## Materials and methods

### Materials

Lipid C12-200 (CAS: 1,220,890–25-4) was obtained from Corden Pharma, Switzerland. 1,2-di-O-octadecenyl-3-trimethylammonium propane (chloride salt) (DOTMA, 890,898), Distearoylphosphatidylcholine (DSPC, 850,365), Cholesterol (Chol, 700000P), 1,2-distearoyl-sn-glycero-3-phosphoethanolamine-N-[amino(polyethylene glycol)-2000]-N-(Cyanine 5) DSPE PEG(2000)-N-Cy5 (Cy5 PEG, 810,891), and 1,2-dimyristoyl-rac-glycero-3-methoxypolyethylene glycol-2000 (DMG-PEG(2 k), 880151P) were obtained from Avanti Polar Lipids, Alabama. Fasted State Simulated Gastric Fluid (FaSSGF), Fasted State Simulated Intestinal Fluid (FaSSIF), and Fed Sate Stimulated Intestinal Fluid was prepared using 3F Powder ™ (Biorelevant©, FFF02) and FaSSGF Buffer concentrate (Biorelevant©, FASGBUF), FaSSIF Buffer Concentrate (Biorelevant©, FASBUF), and FeSSIF Buffer Concentrate (Biorelevant©, FESBUF), respectively. Sodium Taurcholate (CAS: 145–42-6) was purchased from Spectrum ®. siRNA against Glyceraldehyde-3-phosphate dehydrogenase (GAPDH), siGAPDH (Invitrogen™, Silencer™, AM4632), Cy-3 labeled siGAPDH (Invitrogen™, Silencer™, AM4640), and negative control siRNA (siNeg, Invitrogen™, Silencer™ Select Negative Control No. 1 siRNA, 4,390,843) was obtained from ThermoFisher Scientific. siRNA against Hypoxanthine–guanine phosophoribosyltransferase (HPRT), siHPRT was obtained from Takeda Pharmaceuticals had the sequence (Sense strand: GCCAGACUUUGUUGGAUUUGA, anti-sense strand: UCAAAUCCAACAAAGUCUGGCUU). CleanCap®Firefly Luciferase mRNA (5-methoxyuridine) was obtained TriLink™ (FLuc mRNA, L-7202). Quant-it™ Ribogreen RNA Assay kit (Invitrogen™, R11490), TaqMan™ Fast Advanced Cells-to-CT™ Kit (Invitrogen™, A35374), KDalert™ GAPDH Assay Kit (Invitrogen™, AM1639), Pierce™ BCA Protein Assay Kit (Thermo Scientific™, 23,227), PureLink™ RNA Mini Kit (Invitrogen™, 12,183,025), High-Capacity cDNA Reverse Transcription Kit (Applied Biosystems™, 4,368,814), TaqMan™ Gene Expression Master Mix (Applied Biosystems™, 4,369,542), HPRT Taqman primer (Mm03024075_m1), Peptidyl-prolyl cis–trans isomerase B (PPIB) Taqman primer (Mm00478295_m1) and GAPDH Taqman primer (Mm99999915_g1) was obtained from ThermoFisher Scientific. Dulbecco's Modified Eagle Medium (DMEM, 11,965,118), Cell Dissociation reagent (Gibco™, TrypLE™ Express Enzyme (1X), phenol red, 12,605,010), 0.25% Trypsin–EDTA (Gibco™, 25,200,056), penicillin/streptomyocin (Gibco™, 15,070,063), Opti-MEM™ Reduced Serum media (OMEM, Gibco™, 31,985,062), and Phosphate Buffered Saline (PBS, pH 7.4, Gibco™, 10,010,023) were purchased from Life Technologies. Fetal Bovine Serum (FBS, Sigma-Aldrich ™, 12103C-100ML), Mucin from porcine stomach (Sigma-Aldrich®, M2378-100G) was obtained from Millipore Sigma. CellTiter-Fluor™ Cell Viability Assay (G6081), ONE-Glo™ + Tox Luciferase Reporter and Cell Viability Assay (E7120), was obtained from Promega Corporation.

### LNP preparation

LNPs were prepared by microfluidic mixing using the Nanoassemblr™ Ignite™. All Lipids were added in C12-200: Cationic Lipid: DSPC: Chol: DMG-PEG(2 k) (50-x):x:38.5:10:1.5 mol% and the N/P indicated for each experiment, unless otherwise mentioned. siGAPDH or FLuc mRNA was the payload used for in vitro and in vivo studies. Total Flow Rate (TFR) was set to 12 mL/min and Flow Rate Ratio was set to 3:1 (aqueous:organic). Upon LNP preparation by microfluidic mixing, the samples were added into 20 kDa MWCO dialysis cassettes and dialyzed overnight against 100 × vols. of 1 × PBS, followed by concentration using Amicon filtration tubes (30 kDa MWCO) at 2000 g. The lipid concentration was fixed at 2 mg/mL during preparation of different formulations ensuring that all the formulations have the same overall lipid concentration despite differing in lipid composition.

With the base formulation as C12-200: DOTMA: Chol: DMG-PEG(2 k) (30:20:38.5:1.5), fluorescently labeled LNPs were prepared by adding 0.5% Cy5 labeled PEG to 1% DMG-PEG and replacing 25% of unlabeled siRNA with Cy3-labeled siRNA. Using these proportions, 3 types of fluorescently labeled LNPs were obtained with Cy5 labeled PEG, Cy 3 labeled siRNA and a mixture of the two.

### LNP characterization

Routinely, the samples were diluted 100 × in 1 × PBS to be tested for size and 10 × in 0.1 × PBS for surface charge using dynamic light scattering (Malvern Panalytical©, Zetasizer Ultra). Three readings are made for size and zeta potential, standard deviation has been plotted. Encapsulation was assessed using Quant-it™ Ribogreen assay as per manufacturer’s protocol using SpectraMax® i3x (Molecular Devices©). Four readings are taken for the ribogreen assay, standard deviation is plotted.

### Formulation stability assessment in biorelevant media

FaSSGF,FaSSIF, and FeSSIF was prepared according to manufacturer’s protocol. 2% and 5% Mucin was prepared in 1 × PBS. For these experiments, the formulations were concentrated to mimic animal dosing (2 mg/kg of siRNA LNP). The formulation was diluted with different volumes of FaSSIF, FaSSGF, and FeSSIF and incubated at 37˚C, under orbital shaking 280 rpm for 1 h. The formulations were diluted with biorelevant media at 1:1, 1:3, 1:5 (v/v) according to a published study [22]. For mucin experiments, equal volumes of LNP and Mucin was incubated at 37 ˚C, under orbital shaking 280 rpm for 1 h.

### Cell culture

RAW 264.7 cells (Murine Macrophage cell line) and Caco-2 cells (Human Colorectal cancer cell line) was obtained from ATCC. RAW 264.7 and Caco-2 cells are grown in DMEM supplemented with 10% FBS and 1% P/S. The cells were incubated at 37˚C in a 5% CO_2_ environment and subculture by partial digestion with TrypLE Express. Passage numbers 10–20 for RAW 264.7 cells and 30–40 for Caco-2 cells was used for most experiments.

### In Vitro transfection studies

RAW 264.7cells or Caco-2 cells were plated at 10,000 cells/well in a 96 well opaque cell culture plates or standard plates overnight. At the time of transfection, the media was changed to OMEM. Samples were diluted in OMEM and added to the wells. At 48 h, the media was removed, and wells were washed with 1 × PBS before being lysed and analyzed using the KDAlert™ kit for GAPDH knock down and Pierce’s BCA kit for total protein assay according to manufacturer’s protocol. CellTiter-Fluor™ Cell Viability Assay was used for viability assessment for siRNA transfection studies according to manufacturer’s protocol. In some cases, for siRNA studies, toxicity was assessed based on GAPDH protein values when negative control siRNA was used with the same LNP formulation. To measure gene knockdown, transfection was carried out for 24 h instead of 48 h, and TaqMan™ Fast Advanced Cells-to-CT™ Kit was used as per manufacturer’s protocol. For mRNA analysis, after 48-h incubation, ONE-Glo™ + Tox Luciferase Reporter and Cell Viability Assay was performed according to manufacturer’s protocol.

### LNP uptake in RAW 264.7 and Caco-2 cells by flow cytometry and confocal microscopy

RAW 264.7 cells and Caco-2 cells were plated at a density of 10,000 cells/well in 2 collagen-coated plates overnight before proceeding to transfection. An optical polymer 96-well plate (Corning) for uptake imaging and a BioCoat (Collagen I, Corning) plate was used for flow cytometry. LNPs (base, Cy3-siRNA LNPs, Cy5-PEG LNP, Cy3 siRNA + Cy5 PEG LNP) were suspended in OMEM at 0, 0.2, 2, 20, and 200 nM concentration of siRNA for sample preparations. Samples were analyzed at 0, 0.5, 1, 4, 6, and 24 h.

Post-exposure, cells were lifted in 0.25% Trypsin–EDTA after treatment for 5 min and collected in microcentrifuge tubes. The cell suspension was centrifuged at 400G for 5 min at 4˚C and the pellet was resuspended in 250 µL 1 × PBS. Cells were then analyzed using BD Symphony and analyzed using Flowjo v10.7.2. The fluorescence intensities were detected after surveying the channels and selecting the most appropriate wavelengths. 150µL of sample was collected. Cells were gated using forward vs. side scatter plots to exclude doublets and cell debris. Untreated cells were used as negative-staining controls to set a gate for Cy3/5-negative and Cy3/5-positive populations. Data are presented as a percentage of Cy5-positive (Cy5 ( +)) and Cy3-positive (Cy3( +))events.

Confocal microscopy was performed by imaging the wells every 15 min on Zeiss LSM 900 confocal microscope with an incubator attachment module.

### In Vivo studies

Animal protocols were approved by the Institutional Animal Care and Use Committee (IACUC) at Takeda Pharmaceuticals (Cambridge, MA). Female B6/J mice or SKH1 mice (8 week) old was purchased from Jackson Labs, ME. Mice were housed under controlled temperature (25 °C) in 12-h light–dark cycles. Animals were given access to standard diet and water.

For siRNA analysis, SKH1 mice were used. Following 10 h fasting, mice were weighed and dosed at 10 mL/kg, 2 mg/kg of siHPRT LNP, by oral gavage. Food was introduced 6 h after dosing (total: 16 h fasting). The mice were sacrificed at 6 h, 24 h and 48 h after dosing. Esophagus, stomach, duodenum, jejunum ileum, and colon were collected, homogenized to assess for HPRT knock down.

For mRNA analysis, B6/J mice were shaved the previous evening so that the hair does not confound whole body imaging through IVIS (Perkin Elmer, MA). Following 10 h fasting, mice were dosed according to the study design. Food was introduced one hour after dosing. First read out was taken at 6 h after LNP dose [19]. Luciferase expression was be monitored after the IP administration of 130µL D-Luciferin substrate (30 mg/mL). Food was introduced to the animals after imaging (total 16 h fasted). The second read out was taken 24 h after LNP dose. Luciferase expression was monitored after the IP administration of 130µL D-Luciferin substrate (30 mg/mL). At 24 h, the animals were sacrificed, and the organs are excised and confirmed for luminescence using IVIS.

### RT-qPCR

For in vitro experiments, Cells-to-CT™ Kit was followed per manufacturer’s protocol. For in vivo studies involving RT-qPCR analysis, RNA was extracted using PureLink™ RNA Mini Kit as per manufacturer’s protocol. The total RNA solution is diluted to 100 ng/mL with DNAse/RNAse free water, before proceeding with Reverse Transcription using High-Capacity cDNA Reverse Transcription Kit, as per the manufacturer's instructions. C1000 Touch™ Thermal Cycler with 96-Well Fast Reaction Module (Bio-rad, 1,851,196) is used as a Thermocycler. For qPCR, TaqMan™ Gene Expression Master Mix is utilized using Taqman primers for the gene to study. QuantStudio 6 Flex (Applied Biosystems™) is used to carry out qPCR.

### Statistical data analysis

Average and Standard Deviation (STDEV) were plotted for all studies. Prism 8.1.0 was used to perform statistical analysis and generate graphs. For studies where multiple groups were compared, Ordinary one-way ANOVA was implemented to determine statistical significance with Tukey correction. For studies where only 2 groups were compared, unpaired two-tailed Welch’s t test was used to determine statistical significance.

## Results

### Addition of DOTMA to the LNP enhances siRNA encapsulation and In vitro gene silencing

**Fig. 1:**
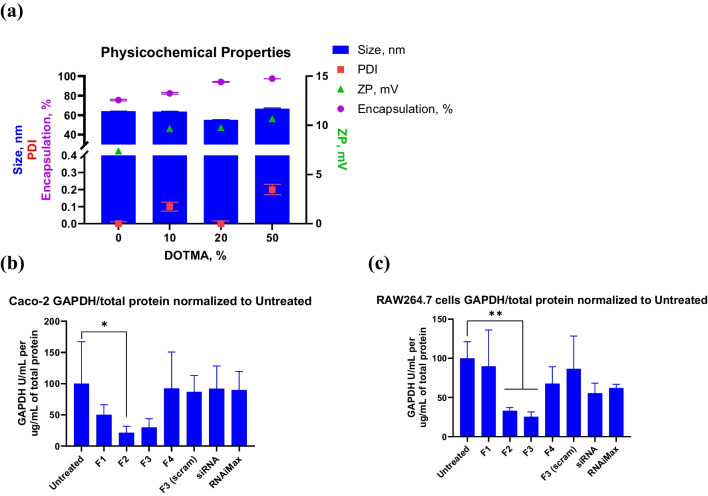
OrD LNPs- Addition of DOTMA to the formulation enhances gene knockdown. **a** Physicochemical properties size (blue bar), PDI (red square), zeta potential (green triangle), encapsulation (purple circle) of all the formulation studied in this experiment. n = 3/4, standard deviation plotted. **b** Caco-2 cells and (**c**) RAW 264.7 cells were incubated with 10 nM siGAPDH encapsulated in the 4 LNP formulations F1 (0% DOTMA), F2 (10% DOTMA), F3 (20% DOTMA), F4 (50% DOTMA), show F2 and F3 to be the more potent amongst the formulation. F3 (scram)acts as a control for toxicity of the formulation. n = 5, standard deviation plotted. *p < 0.05, **p < 0.001

Formulation development is crucial to stabilize the LNP in a biological environment. This led us to explore the addition of a cationic lipid to the base formulation C12-200: Cationic Lipid: DSPC: Chol: DMG-PEG(2 k) (50-x):x:38.5:10:1.5 mol%, where x was varied from 0–50 (Table 1). The rationale to introduce a cationic lipid was to improve stability of the LNP core and enable it to resist biorelevant media mediated destabilization. DOTMA was chosen as the preferred cationic lipid since it does not have hydrolysable ester linkages. Most ester linkages are more susceptible to degradation especially in the GIT [23, 24]. Systematic addition of DOTMA showed an increase in surface charge and an improvement in encapsulation efficiency (Fig. 1.a). Caco-2 and RAW 264.7 cells were used to test in vitro efficacy of these formulations (Fig. 1.b and c). 20% DOTMA formulation (F3) with scrambled (scram) siRNA sequence was used as a vehicle control to confirm that the knock down is specific to siRNA sequence with minimal activity from the carrier LNP. 10% and 20% DOTMA formulation showed the best target gene knock down activity in both Caco-2 and RAW 264.7 cells (Fig. 1.b and c respectively) compared to other tested formulations. Interestingly, 50% DOTMA formulation did not demonstrate any in vitro activity, possibly due to endosomal entrapment of the LNP. This trend underscores the important balance in the ratio of ionizable to permanently charged cationic lipid in facilitating the endosomal escape of the cargo and influencing in vitro activity. The LNP containing scrambled siRNA as well as naked siRNA controls did not demonstrate any activity in vitro as expected.

**Table 1:** LNP formulations encapsulating siRNA evaluated for this experiment

Formulation	Mol%				
	DOTMA	C12-200	Cholesterol	DSPC	DMG-PEG
F1	0	50	38.5	10	1.5
F2	10	40	38.5	10	1.5
F3	20	30	38.5	10	1.5
F4	50	0	38.5	10	1.5

### Addition of DOTMA to the LNP enhances mRNA encapsulation and in vitro gene expression

**Fig. 2:**
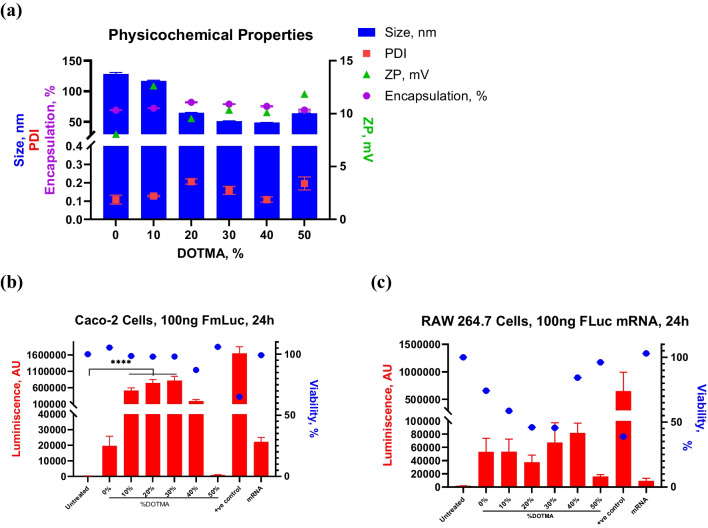
OrD LNPs- Combination of DOTMA and C12-200 in the formulation enhances gene expression. (**a**) Physicochemical properties size (blue bar), PDI (red square), zeta potential (green triangle), encapsulation (purple circle) of all the formulation studied in this experiment. n = 3/4, standard deviation plotted. (**b**) Encapsulation of the mRNA also enhances with the addition of DOTMA. (**c**) Caco-2 cells and (**d**) RAW 264.7 cells show enhances transfection with the addition of DOTMA in the formulation, however maximum DOTMA (without any C12-200) shows poor gene expression. n = 5, standard deviation plotted ****p < 0.0001. MessengerMax™ is used as the positive control

We further explored the robustness of our formulation optimization approach and demonstrate its capability to deliver mRNA as an alternative nucleic acid that requires cytoplasmic exposure for its activity. mRNA as a therapeutic nucleic acid has been extensively explored for delivery using LNPs for transient expression of a protein of interest [25]. Unlike siRNA, mRNA molecules are larger, single stranded and more susceptible to nuclease degradation. The payload versatility of our formulation approach was assessed by utilizing FLuc mRNA as the model payload. A similar stepwise increasing concentration of DOTMA to replace ionizable lipid in the LNP formulation was explored (Table 2**).** Physicochemical properties indicated an increase in charge with the increase in relative DOTMA concentration (Fig. 2a). 20% DOTMA containing LNPs showed the highest encapsulation efficiency with downward trend at higher concentrations, though all formulations exhibited around 70% mRNA encapsulation (Fig. 2a). The addition of DOTMA also translated to effective protein expression in vitro, with performance trend similar to that observed for siRNA DOTMA LNPs (Fig. 2b and c). Interestingly, mRNA-LNP formulations caused loss in viability in RAW 264.7 cells while still showing mRNA expression. We observed that with increasing concentration of permanently charged lipid from 0–30%, the cell viability declines but improves at higher DOTMA concentrations. We also noticed that the expression remains almost the same despite the loss in viability. This observation is specific to RAW 264.7 cells, Caco-2 cells dosed with the same mRNA-LNP formulation do not show any loss of viability. A recent publication observed that C12-200 containing LNPs with fLuc mRNA was amongst the highest expressing formulation and demonstrated higher inflammation in RAW 264.7 cells [26]. The observed inflammation was linked to endosomal damage, crucial for endosomal escape and, subsequently, for effective mRNA expression. In an inflamed state, macrophages often adopt an M1 phenotype, potentially leading to increased uptake of LNPs [27]. The decreased viability in RAW 264.7 cells could be attributed to phenotype-associated excessive phagocytosis, lipid accumulation and subsequent cationic lipid-dependent toxicity. Additionally, our data suggest that the combination of C12-200 with up to 30% DOTMA may lead to differential toxicity, potentially influenced by differences in uptake or endosomal escape mechanisms. This warrants further investigation to clarify these mechanisms. While no discernable differences in mRNA expression was observed between 10–30% DOTMA, the 50% DOTMA formulation showed very poor activity in both cell lines and this observation was consistent with the trend for siRNA loaded LNPs (Fig. 1b and c).

**Table 2:** LNP formulations encapsulating mRNA evaluated for this experiment

Formulation	Mol%				
	DOTMA	C12-200	Cholesterol	DSPC	DMG-PEG
FM1	0	50	38.5	10	1.5
FM2	10	40	38.5	10	1.5
FM3	20	30	38.5	10	1.5
FM4	30	20	38.5	10	1.5
FM5	40	10	38.5	10	1.5
FM6	50	0	38.5	10	1.5

### Addition of DOTMA improves stability of the LNP in FaSSIF

**Fig. 3:**
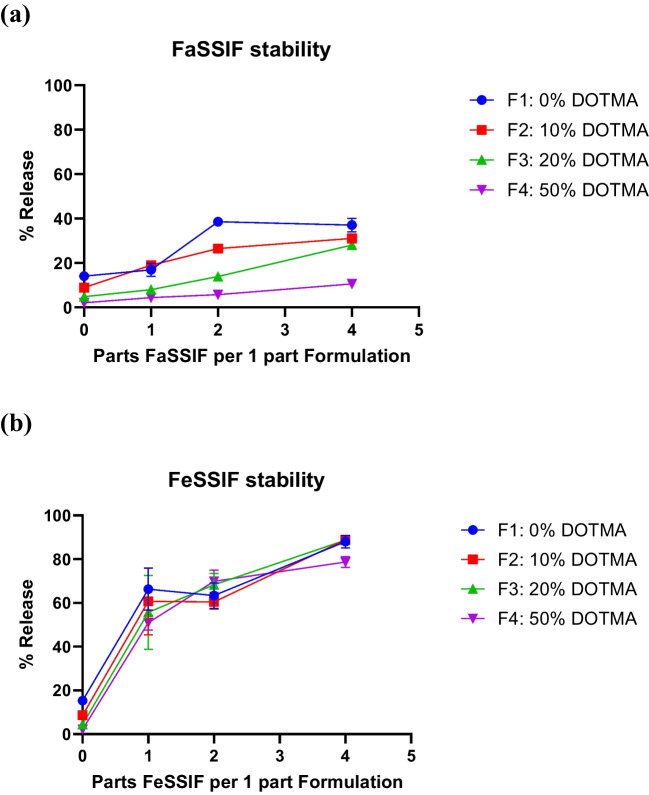
Stability of the LNP at different FaSSIF and FeSSIF dilutions after 2 h incubation. Comparing the stability of formulations F1 (0% DOTMA), F2 (10% DOTMA), F3 (20% DOTMA), F4 (50% DOTMA) through siRNA release at different dilutions of (**a**) FaSSIF and (**b**) FeSSIF indicating improved stability of the formulations in Fasted Intestinal Stimulated Media with increased DOTMA content. n = 4, standard deviation plotted

One of the key challenges of oral drug delivery is the stability of the formulation in the GIT microenvironment. The GIT microenvironment has extreme pH variations and contains bile salts, lipases, nucleases, and other enzymes that can have detrimental impact on LNP integrity. Bile salts particularly are responsible for fat solubilization and are mainly present in the intestines. So it is critical to assess stability of the LNP formulation in biorelevant media that is representative of pH and bile salt composition of the intestines. Fasted simulated intestinal fluid (FaSSIF) and fed state simulated intestinal fluid (FeSSIF) depict the two states of intestinal media, differing in pH and sodium taurocholate (type of bile salt) concentration amongst other salts. FaSSIF has a pH of 6.5 with a sodium taurocholate concentration of 3 mM, while FeSSIF has a pH of 5 and a sodium taurocholate concentration of 15 mM [28]. The impact of addition of DOTMA into the LNPs on their stability was ascertained by measuring the siRNA release profile when LNPs were co-incubated in biorelevant media at different ratio. The LNPs were incubated with FaSSIF for 2 h at different dilutions to mimic LNPs dilution as they pass through the GIT (Fig. 3**.a**). Data depicts that LNP stability in the biorelevant media improved with increasing concentration of DOTMA, with 50% DOTMA containing formulation being the most stable amongst the ones studied. The 4 formulations that were incubated with FaSSIF at 1–1 dilution were checked for RNA release using gel electrophoresis and the obtained data corroborates with the observation made from Ribogreen assay (**SI. **Figure 1). The stability of the LNPs was further challenged by incubating them in FeSSIF (Fig. 3**.b**). As expected, due to the higher sodium taurocholate concentration, all the LNPs were destabilized and released the RNA at a much lower FeSSIF dilution when compared to corresponding data with FaSSIF incubation. Poor stability of the LNPs in FeSSIF highlights the limitation of the formulations to overcome bile salt mediated destabilization.

These experiments elucidate that although elevated concentrations of DOTMA augments stability in fasted intestinal media, the inclusion of C12-200 remains crucial for robust in vitro efficacy. Consequently, 20% DOTMA would be optimal in balancing stability and efficacy. The 20% DOTMA formulation was designated as our candidate Orally Delivered (OrD) LNP and was subjected to further evaluation in biorelevant media and in vivo studies.

### 20% DOTMA formulation (OrD LNP) explored for stability in gastric and intestinal fluid conditions and compatibility with mucus

**Fig. 4:**
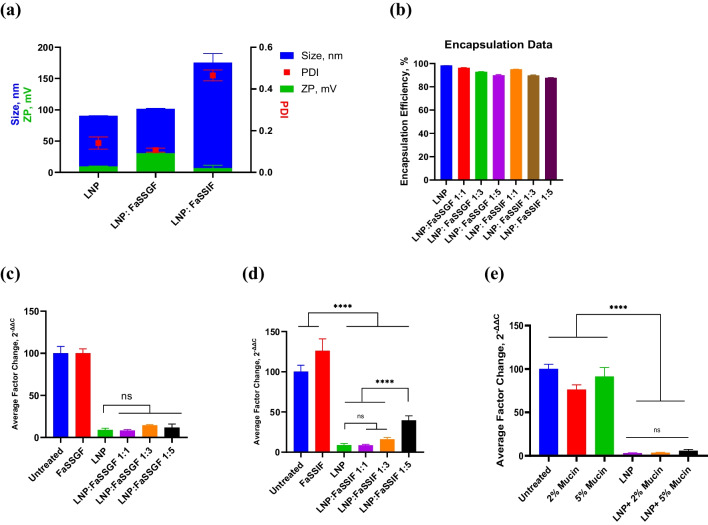
OrD LNP stability under mouse GI conditions of volume dilutions of 1:1 to 1:5 dilution in Gastric Fluid and in Intestinal Fluid for 1 h at 37 °C. OrD LNPs concentration was chosen equivalent to 2 mg/kg dose (0.2 mg/mL) (a) Representative Physicochemical data shows larger distribution in FaSSIF (1:5 dilution) due to the bile micelles and higher positive charge in Gastric media (1:3 dilution) due to ionization of positive charge. n = 3, standard deviation plotted (b) Encapsulation Data shows good stability after 1 h of incubation at 37 °C. n = 4, standard deviation plotted. GAPDH mRNA Expression in RAW 264.7 cells after incubation in (c) FaSSGF and (d) FaSSIF for 1 h at 37˚C (f) HPRT mRNA Expression in RAW 264.7 cells after LNP incubation in 2% and 5% Mucin shows retention of activity after incubating in GI media and mucin. n = 5, standard deviation plotted, *ppib* is used as the endogenous control, **** p < 0.0001

Structural integrity and physicochemical stability of a formulation in the biomimetic conditions is of paramount importance to retain its potency in vitro as well in vivo. The OrD LNP formulation was further explored for stability and subsequent potency in vitro after preincubation in biorelevant media at different dilutions, covering a wide range of mouse GI volumes and dilutions in different compartments of the GIT [22]. The representative physicochemical data (Fig. 4a) indicates a higher positive charge for LNPs in FaSSGF- the fasted gastric simulated fluid with a pH of 1.6. The higher positive charge is expected, as the ionizable lipid will assume positive charge at pH lower than its pKa of 6.96 [29]. Under FaSSIF incubation conditions, the size data from LNPs is difficult to discern since FaSSIF solution itself shows particulate content (DLS data), possibly due to the micelles formed by sodium taurocholate (data not shown). The larger particle size may represent a combination of LNPs, Sodium taurocholate micelles, and perhaps destabilized mixed micelles [30]. Regardless, the encapsulation data shows the stability of the LNPs within the biorelevant media (Fig. 4b).

To ascertain if stability translated to potency, the OrD LNP was preincubated with FaSSGF and FaSSIF were assessed for their target gene knockdown in RAW 264.7 cells (Fig. 4c and d). LNPs that were incubated in FaSSGF at different concentrations exhibit the same potency as naïve LNPs, indicating that the formulation is not impacted upon incubation in the gastric media. The potency of the formulation is retained after incubation is FaSSIF at all dilutions studied. A slight loss in potency was observed at the highest dilution (1:5) with FaSSIF, despite being more potent than untreated samples.

Additionally, the effectiveness of the LNP after being incubated with equal volumes of 2% and 5% mucin was tested for gene knockdown in RAW 264.7 cells (Fig. 4e). LNPs retained their potency after incubation with mucin. Overall, this data confirms the OrD LNP formulation retained their configuration and potency in GIT biorelevant media and mucin, justifying in vivo investigations*.*

### OrD LNP uptake studied in RAW 264.7 and Caco-2 cells using flow cytometry and confocal imaging

It is pertinent to study the kinetics of OrD LNP uptake in relevant cell lines. This will help to understand the therapeutic dosing and potential spatiotemporal intricacies for this formulation. Thus, to determine uptake of OrD LNP, flow cytometry and confocal microscopy were used in tandem.

**Fig. 5:**
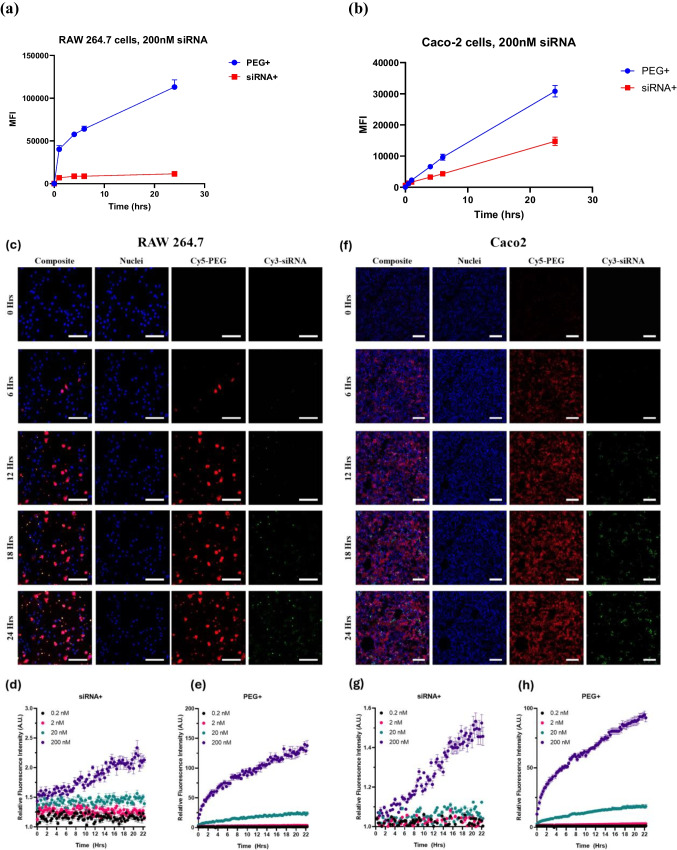
Uptake Kinetics of OrD LNP in RAW 264.7 cells and Caco-2 cells. Dually labeled OrD LNPs studied at a dose of 200 nM siRNA over 24 h in (**a**) RAW 264.7 cells and (**b**) Caco-2 cells. Confocal microscopy images of (c) RAW 264.7 cells, quantified as (d) siRNA + and (e) PEG + , and (f) Caco-2 cells, quantified as (g) siRNA + and (h) PEG + at 24 h and with 200 nM concentration of siRNA corroborating with the confocal microscopy data

Cells were analyzed for siRNA + (Cy3-labeled) and PEG + (Cy5-labeled) using filters in flow cytometry that would detect signals of the siRNA cargo and the LNP respectively, within the cells. Doses ranging between 0 to 200 nM were tested over 24 h of incubation of the cells with the LNP suspension (Error! Reference source not found.**-c).** At 200 nM siRNA dose, both RAW 264.7 and Caco-2 cells exhibit rapid uptake of LNPs within the first 6 h (Fig. 5. a and b). Furthermore, the signal in RAW264.7 cells is nearly 6 times higher than in Caco-2 cells at the 6 h timepoint indicating the differences in LNP processing. It was interesting to note, the PEG-Cy5 signal peaks prior to the siRNA-Cy3 signal and this was especially prominent in Caco-2 cells. There seems to be a time lag in the signal between the PEG and siRNA and some difference in signal intensities between the two cell types. Confocal microscopy data in RAW 264.7 cells (Fig. 5. c, d and e) and in Caco-2 cells (Fig. 5f, g and h) corroborates with the flow cytometry data.

### In vivo study with OrD LNP-siRNA shows gene knock down in GIT

**Fig. 6:**
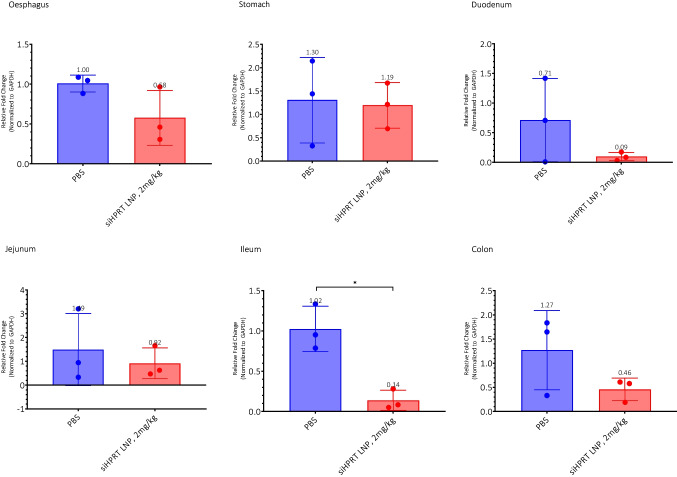
Oral delivery of siHPRT OrD LNP (C12-200: DOTMA: DSPC: Chol: DMG-PEG(2 k) 30-:20:38.5:10:1.5 mol% shows gene silencing in the GIT. Female SKH1 mice, HPRT gene silencing data after 48 h after 2 mg/kg siHPRT-LNP dosing in different GI organs. n = 3, standard deviation plotted, p* < 0.05

LNPs were tested for in vivo efficacy to knock down endogenous HPRT gene (Fig. 6). 2 mg/kg of siHPRT encapsulated in OrD LNP was administered in SKH1 female mice after overnight fasting. Food was introduced 6 h after dosing. Animals were necropsied at 24 h and 48 h. All the GI organs were extracted, and mRNA levels were quantified by RT-qPCR using GAPDH as the endogenous gene, for 24 h and 48 h timepoints.

While HPRT knock down wasn’t observed at 24 h (SI Fig. 3), after 48 h of dosing, the trend favors gene knockdown with 84% knockdown observed in the ileum (Fig. 6).

### In vivo studies with OrD LNP-mRNA shows luminescence from the upper GIT

**Fig. 7:**
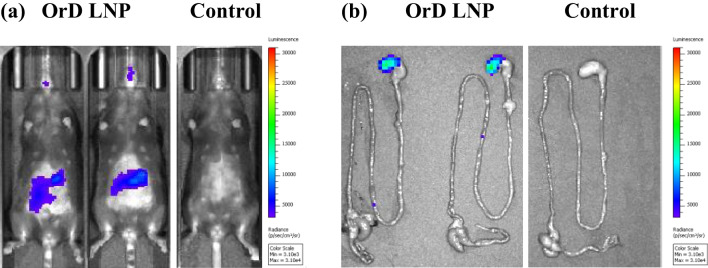
Oral delivery of 2 mg/kg mRNA OrD LNP (C12-200: DOTMA: DSPC: Chol: DMG-PEG(2 k) 30-:20:38.5:10:1.5 mol%) mediated gene expression in B6/J female mice 6 h after dosing. IVIS images for (**a**) whole body and (**b**) necropsied GIT showing luminescence localized in the upper GIT

One of the main aims of this research was to develop a platform formulation that could work for a variety of different nucleic acids. We have used FLuc mRNA as a model mRNA to demonstrate the versatility of OrD LNPs. OrD LNPs encapsulated with FLuc mRNA was tested in vivo to qualitatively study the potency. 2 mg/kg of FLuc mRNA encapsulated in the OrD LNP was administered orally. The size (74.66 ± 0.2 nm), polydispersity index (0.21 ± 0.00), surface charge (+ 12.18 ± 0.27 mV) and encapsulation of 94.2 ± 0.36% indicates a stable LNP formulation for dosing. PBS was administered orally to the control mice. Luminescence was captured using IVIS on the whole-body imaging and on harvested organs for the OrD LNP treated and control groups. IVIS data indicates luminesce in the GIT of animals treated with OrD LNPs in comparison to the control animals. Upon organ isolation and subsequent IVIS, luminescence was confirmed to be localized to the upper GIT (non-glandular portion of the stomach) of the animals treated with OrD LNPs (Fig. 7). The signal accumulation in the upper GIT may be due to the assumption of a positive charge on the LNP in relation to the pH. Further studies must be performed to understand tissue and cell specificity of the OrD LNPs.

## Discussion

As evidenced by contemporary research, the success of oral delivery of nucleic acid lies in the ability of the vehicle to maintain its stability within the GIT whilst being potent to transfect the cell and release the payload in the cytosol. In this research, the addition of a cationic lipid was explored as a formulation component and was systematically assessed for stability in biorelevant media. Additionally, its efficacy in vitro and in vivo for two different RNA payloads demonstrates the versatility of the formulation.

DOTMA as the permanently charged cationic lipid was added to the formulation to maintain permanently cationic core and facilitate better nucleic acid complexation. DOTMA has been extensively used, either on its own or in combination with a helper lipid to carry plasmid DNA [31–35]. In fact, DOTMA provided better in vivo transfection than DOTAP despite the two differing only in the linkage bonds [33]. Ether bond present on DOTMA would make it less susceptible to hydrolysis in the GIT. In the present work, we systematically replaced C12-200 with DOTMA in the formulation When tested in vitro in RAW 264.7 cells and Caco-2 cells, the addition of DOTMA showed potency both with siRNA and mRNA, when compared to the formulations without DOTMA. Interestingly, 50% DOTMA formulation that had no ionizable lipid C12-200, lacked potency. This underscores the importance of the presence of ionizable lipid in facilitating endosomal escape. It will be interesting to study the impact of different ionizable lipids on the efficacy of the formulation when dosed orally.

This study utilized several biorelevant media—FaSSGF, FaSSIF, and FeSSIF—to simulate different microenvironments within GIT and these simulated conditions were critical for assessing the stability and efficacy of formulations as they transit from the stomach to the intestines. FaSSGF, mimicking the fasted gastric state, has a pH of 1.6 and contains minimal bile salts and phospholipids. FaSSIF models the fasted intestinal state at a pH of 6.5, with higher concentrations of bile salts and phospholipids, while FeSSIF represents the fed intestinal state with a pH of 5 and the highest concentrations of these components. The study adjusted LNP concentrations and media dilutions to replicate physiological transitions within the GIT, although each media was considered in isolation.

Bile salts form micelles that solubilize lipids to aide fat digestion and subsequent absorption [36–38]. Several studies have been performed exploring fat solubilization using surfactants such as sodium taurocholate (bile salt) [30, 39, 40]. There is a correlation between the concentration of lipid used and the bile salt/surfactant present. There is also a proposed means of solubilization in which case, surfactants interact with lipidic structures (liposomes) forming mixed micelles as the surfactant concentration increases, partitioning into the bilayers, before completely obliterating the liposome into smaller micelles [30]. This also underscored the importance of the use of different biorelevant dilutions to show the concentration relation between the LNP and the bile salts. OrD LNP demonstrated success in overcoming bile salt in fasted media. However, the formulation showed limited stability in fed state media with higher bile salts, revealing the need to fast the animal to control bile salt mediated destabilization corroborating with some previous studies [18, 41]. The OrD LNP stability in the biorelevant media was pertinent in designing our in vivo studies under fasted conditions and temporal spacing of food availability in animal model.

Porcine mucin was also used to analyze the efficacy of OrD LNPs, thus aligning experimental conditions with in vivo environments. We mixed mucus with the OrD LNP, tested in vitro efficacy to demonstrate their compatibility. Use of specialized in vitro models such as a transwell system with mucus added on the donor side and PBS on the acceptor side would further aid in understanding the ability of the oral delivery systems to traverse through the mucosal barrier and delivery the payload successfully [42]. It is also important to point out that our study did not consider enzymes in the biorelevant media. Since a previous study depicted pepsin mediated aggregation [18], enzymes could be considered to understand its impact on efficacy. Further protection of the OrD LNPs against other degradants could be developed by engineering a composite system such as NiMOs or polymeric coating [41, 43].

OrD LNPs were assessed for uptake kinetics in RAW 264.7 and Caco-2 cells using flow cytometry and confocal microscopy. Fluorescently labeled siRNA and PEG were used for these experiments. The cells exhibit consistent uptake of these formulations for the initial six hours, after which the rate appears to diminish. This was more pronounced at higher concentrations of the LNP and can be seen more clearly in Caco-2 cells than in RAW 264.7 cells perhaps due to either cell volume or differences in metabolism [44]. A discernible lag between the PEG and siRNA fluorescence is observed, likely indicating differential intracellular processing times. In the data presented by Khare et al. C12-200 LNPs with Cy5 labeled siRNA indicated slow uptake at earlier timepoints of 2 and 4 h while noticeable increase in siRNA uptake was observed 24 h post transfection on neural cells [45]. Furthermore, the LNP first must enter the cell, then get processed in the endosome that would lead to the endosomal release of the payload for cytoplasmic exposure. Based on the general steps itself, the LNP has fewer steps than the siRNA itself that would justify the differences in the way the two modalities are processed during uptake and beyond.

With in vitro analysis depicting positive outcome, the OrD LNP was tested in vivo*.* OrD LNP encapsulating siRNA against *HPRT* (siHPRT) is studied. While statistically significant knock down was observed in the ileum after 48 h, the levels were sub-par in rest of the GI organs. *HPRT* is an endogenous gene, and oftentimes endogenous housekeeping genes may not be the best targets to study naïve animal knockdown studies, since they are abundantly present and can be compensated by other mechanisms. The lower level of knockdown in vivo in housekeeping gene *GAPDH* has been studied previously to show sub-par efficacy as well [18]. siRNA dose could be another reason for lower knockdown efficiency, perhaps 2 mg/kg is insufficient for overwhelmingly positive response in vivo. In our study, the dose was limited by LNP processing through ultrafiltration. Beyond a point, ultrafiltration via centrifugation requires significant time to concentrate the LNPs. Excessive G-force could destabilize the LNPs due to which doses beyond 2 mg/kg were not attempted. One way to concentrate the LNPs would be by using TFF. Additionally, to increase the local concentration of the LNP in the intestines, duodenal catheterized mice could be used. The other factor that could impact knock down is the time elapsed after dosing. 48 h, 72 h has been routinely used for most siRNA studies [46, 47]. However, when administered via the oral route, the kinetics of the particles is very different when compared to parenteral route. This is because there is no loop for the LNPs to travel through in the GIT as is the case for parenteral delivery. Adding to this complexity, enterocytes in mice have a turnover rate of 2.81 days [48]. This should be considered when designing experiments assessing PK and PD response to the oral delivery of siRNA. Lastly, parts of each GI organ were assessed for gene knock down. There is a high likelihood that the uptake of the nanoparticles is not uniform in the GIT and this could potentially introduce sampling limitations where measuring gene knock down efficacy. A potential method for overcoming this would be to perform biodistribution by stem-loop PCR on tissues at the different timepoints after the administration of siHPRT-LNP [49, 50].

Additionally, through a limited proof of concept experiment we demonstrated that OrD LNPs encapsulating mRNA successfully led to luminescent signal in the GIT. The signal signifies not only successful delivery of the mRNA in the cytoplasm but also the successful intracellular translation in the GIT. This depicts the versatility of OrD LNP as a platform. The accumulation of signal from the OrD LNPs in the upper GIT indicate their potential utility for local or systemic delivery in the upper GIT. Majority of nutrient absorption takes place in the small intestines. There are several diseases such as gastroparesis, celiac disease, Crohn’s disease that mainly affect the upper GIT. Some of these diseases have local manifestations in terms of reduced mucosal lining and the generation of a chronic wound. Further assessment of these formulations could explore their utility in systemic delivery through the oral route as well as explore for local delivery of the nucleic acid payload in the upper GIT. While the signal is quantifiable, there is an opportunity to improve. Additional experiments can be planned to improve the signal by increasing the dose, tweaking the dosing regimen or using duodenal catharized animals to reduce the loss of the LNPs in the stomach.

The two payloads siRNA and mRNA used in this research are very different in terms their molecular structure, the way they engage with the RNA machinery, and their overall therapeutic outcomes. It is interesting to note that same OrD LNP formulation can be used for both payloads with improved efficacy when compared with the other formulations studied. Platform technology could aid in the development of combinatorial therapy where both payloads are utilized simultaneously, reducing the development timelines. This is crucial for pleotropic diseases which could utilize both siRNA and mRNA for therapy. From a drug development perspective, this is an essential feature that could expedite formulation development enabling translation from the lab bench to the clinic.

Furthermore, OrD LNP has the potential to start a new era of nucleic acid therapeutics, making medicines easier to administer, ensuring higher patient compliance rates and reducing the overall burden on healthcare. Further research in the area could improve the base formulation to demonstrate local and/or systemic delivery of the payload.

## Conclusion

Our research elucidates a distinct and innovative perspective, emphasizing the imperative consideration of the route of administration during the formulation development process. Notably, administering a labile molecule via the oral route presents substantial challenges. We have demonstrated a methodological approach that involves the systematic integration of DOTMA to develop an Oral Delivery Lipid Nanoparticle (OrD LNP). This formulation has been depicted to maintain stability within the gastric environment and under fasted intestinal conditions. Furthermore, it preserves its efficacy after exposure to biorelevant media and demonstrates effective transfection capabilities within the gastrointestinal tract for both siRNA and mRNA. This study not only advances our understanding of formulation science but also underscores the critical role of thoughtful design in pharmaceutical development.

## Supplementary Information

Below is the link to the electronic supplementary material.

Supplementary file1 (DOCX 875 KB)

## Data Availability

All data analyzed during this study are included in this published article [and its supplementary information files].
