# Supplementary material for: Oral delivery of stabilized lipid nanoparticles for nucleic acid therapeutics
Source: Drug Deliv Transl Res. 2024 Sep 19;15(5):1755–69. doi: 10.1007/s13346-024-01709-4 (PMC11968485; doi:10.1007/s13346-024-01709-4)
Supplement: Supplementary file 1 — Supplementary file1 (DOCX 875 KB) [file 13346_2024_1709_MOESM1_ESM.docx]

**Supplemental Information**

**Supplemental Figures**

**Column 1: LNP**

**Column 2: LNP: FaSSIF (1:1)**

**Column 3: LNP+triton**

**
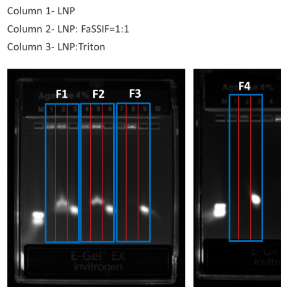
**

**Supplemental Figure 0:** Gel Electrophoresis data for F1 (0% DOTMA), F2 (10% DOTMA), F3 (20% DOTMA), F4 (50% DOTMA) through siRNA release at 1:1 dilution with FaSSIF to depict the stability of F3 and F4.

| 1. **RAW 264.7 cells Gating Strategy**   **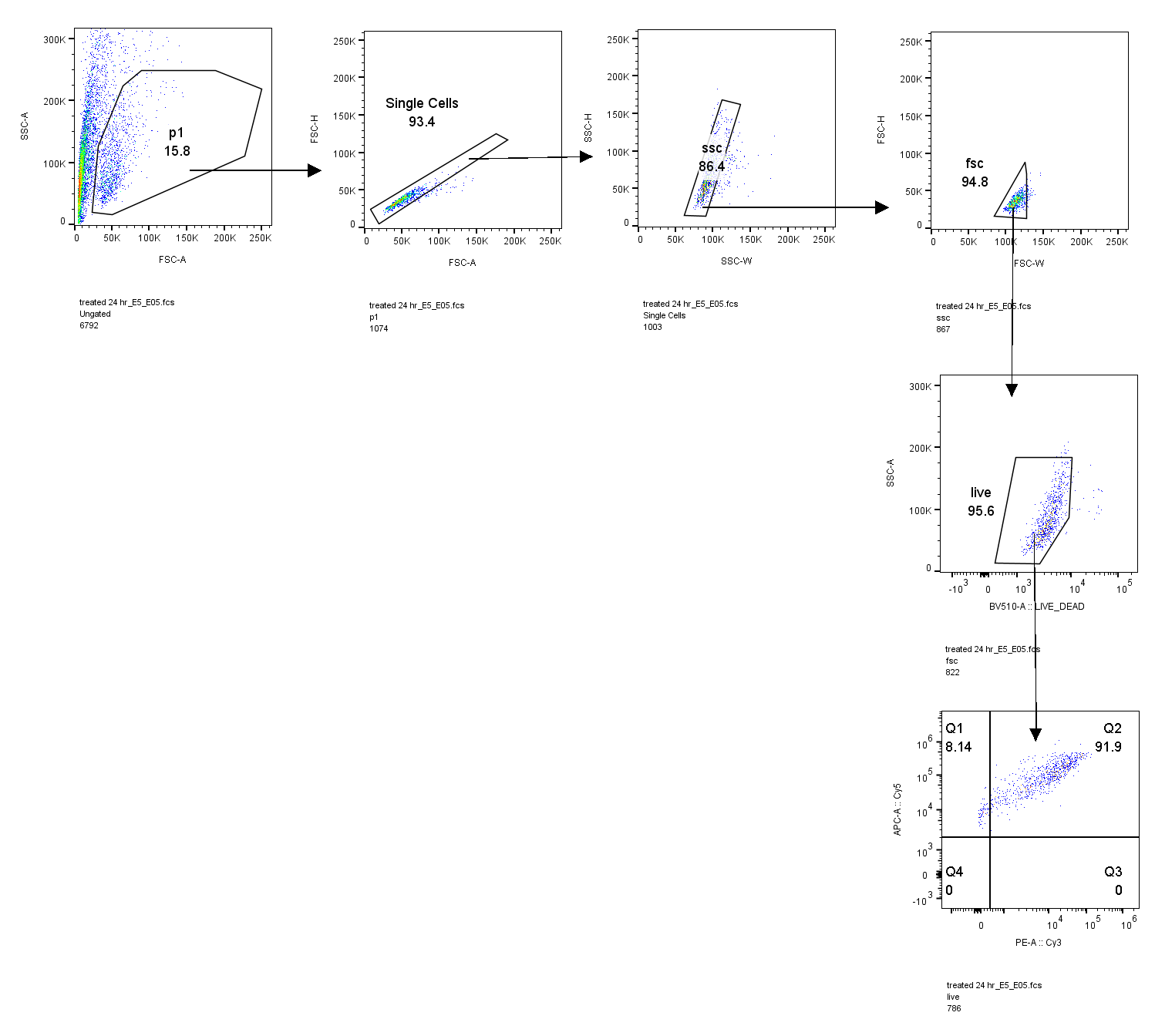**  **Caco-2 cells Gating Strategy**  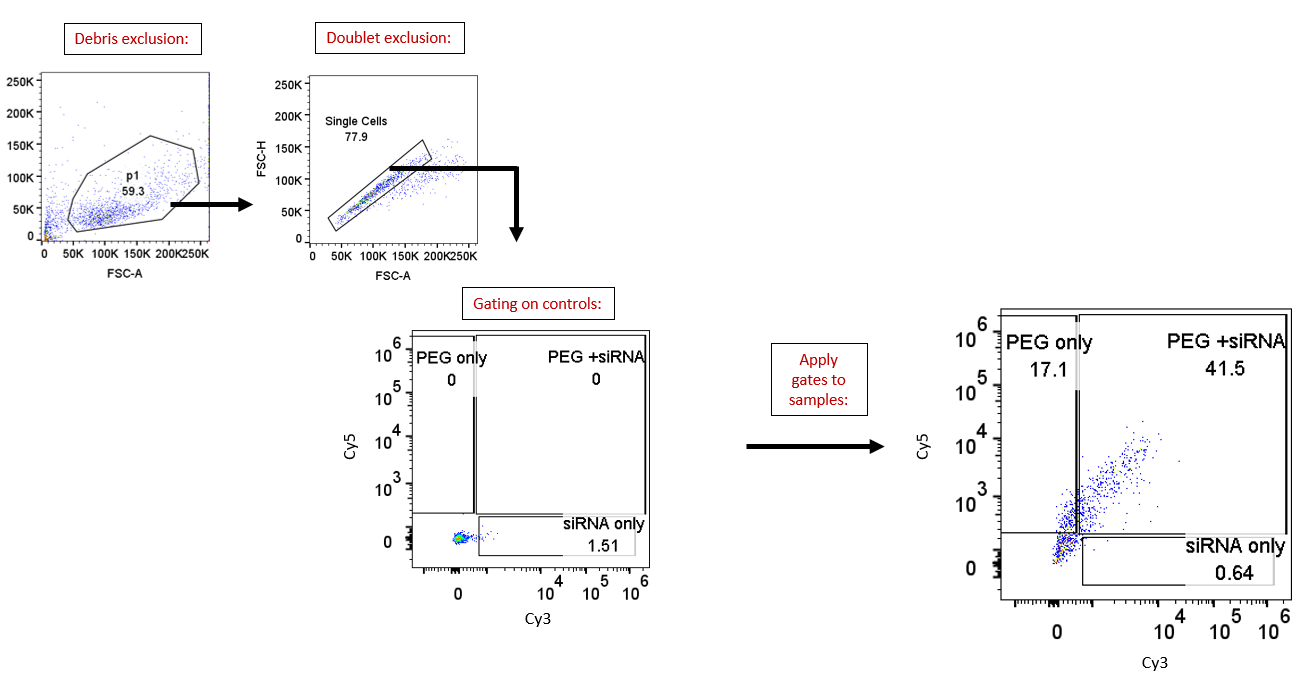 |
| --- |
| **(b)**   |
| **(c)**   |

**Supplemental Figure 2:** Uptake kinetics of OrD LNP in RAW 264.7 cells and Caco-2 cells. (a) Flowcytometry gating strategy for RAW 264.7 and Caco-2 cells. Flowcytometry analysis showing appropriate signals in different concentrations and timepoints for (b) RAW 264.7 cells and (c) Caco-2 cells.

|  |
| --- |

**Supplemental Figure 3:** Oral delivery of siHPRT OrD LNP doesn’t show gene silencing in the GIT after 24h. Female SKH1 mice, HPRT gene silencing data after 24h
